# Person- and People-Centered Integrated Health Care for Alcohol Dependence – Whether It Is Real in the Present Moment

**DOI:** 10.3389/fpubh.2016.00264

**Published:** 2016-11-29

**Authors:** Mirjana Jovanovic, Marko Antunovic

**Affiliations:** ^1^Department for Addiction and Dual Disorders, Clinical Center, Psychiatrist/Psychiatric Clinic, Kragujevac, Serbia; ^2^National Poison Control Center, Military Medical Academy, Belgrade, Serbia

**Keywords:** alcohol addiction, insurance, person integrated health care, harmful use of alcohol, social costs

## Abstract

Alcohol continues to occupy a leading position in Europe as a popular substance of abuse. According to WHO sources together with cigarette smoking and obesity, alcohol is a major cause of preventable diseases. Harmful use of alcohol is one of the main factors contributing to premature deaths and disability and has a major impact on public health. The consequences of alcohol use on human health are enormous. Additionally, alcohol use can have harmful effects that do not directly affect person who consumes alcohol (e.g., fetal alcohol syndrome violations that are related to alcohol use, etc.). It is well known that the harmful effects and consequences of alcohol use (e.g., acute and chronic illness, injuries in fights, at the workplace, in traffic, violent behavior, and death) create a great burden for the economic development of society. Persons who have been diagnosed with alcoholism and currently drinking have a less chance to achieve a life insurance cover. On the contrary, recovering alcoholic with a significant abstinent period can get a good life insurance quote. The abstinence of a year or 2 is usually enough for a person to get an average price of life insurance. Furthermore, new consequent relapses could also be considered as potential aggravating factor to accomplish this kind of financial benefits. So far, the research (and interventions) focused on the effects on the population level, such as the increase in taxes, advertising bans, and the implementation of laws that prevent the use of alcohol in traffic. However, it seems that the problem may be viewed at the individual level. The models of the treatment should be designed according to the needs of the individual. These models should incorporate not only the reduction of alcohol intake but also the path to abstinence. The plan should take into account the different (individual) needs for treatment, with regard to the degree of alcohol dependence and health status and also include the needs of the family, community, and broader society.

Alcohol continues to occupy a leading position in Europe as a popular substance of abuse. Together with cigarette smoking and obesity, alcohol is a major cause of preventable diseases. Although alcohol consumption is relatively stable per capita, an increase was observed in North and Eastern Europe, while a decrease in consumption has been observed in countries where wine is traditionally consumed (1).

Alcohol abuse and alcohol dependence are two entities that have been included in the previous Diagnostic and Statistical Manual of Mental Disorders (DSM) IV classification. However, in the DSM V classification, these disorders are part of a continuum called alcohol use disorders. These disorders are classified as mild, moderate, and severe, depending on fulfilled criteria for diagnosis (2).

As WHO experts concluded, alcohol dependence is one of the world’s leading risk factors for morbidity, disability, and mortality. Harmful use of alcohol is the root of more than 200 diseases and injuries described in the ICD-10 classification (3).

Harmful effects are proportionate to the amount of alcohol consumed. The consequences become more drastic as the amount of alcohol increases. The largest number of dangers to health and mortality come from heavy drinking. About 80% of deaths of men associated with alcohol use are the consequences of heavy drinking, and as much as 67% of deaths of women. Heavy drinking means consuming at least 60 mg/day, of ethyl alcohol for men, and 40 mg/day for women (4).

By “Global status report on alcohol and health” of World Health Organization (5), alcohol consumption leads to 3.3 million or 5.9% of lethal outcomes per year (5). The number is greater than mortality from HIV/AIDS, which is 2.8%, violence which is 0.9%, and tuberculosis which is 1.7%. Additionally, 5.1% of global costs associated with repercussions of alcohol use are spent on diseases and injuries which are a direct consequence of alcohol use [measured in disability-adjusted life years (DALYs)] (5).

The consequences of alcohol use on human health are enormous. Additionally, alcohol use can have harmful effects that do not directly affect the person who consumes alcohol (e.g., fetal alcohol syndrome, violations that are related to alcohol use, etc.).

Also, the harmful effects and consequences of alcohol use (e.g., acute and chronic illness, injuries in fights, at the workplace, in traffic, violent behavior, and death) create a great burden for the economic development of a society.

In Europe, the costs associated with alcohol abuse are around €155.8 billion (6). Alcohol is one of the most important causes of mortality and also a significant devastating factor when it comes to individual and general social welfare. The family also suffers numerous negative effects that result from alcohol abuse.

In addition, alcohol abuse significantly affects the ability to work, both with respect to performance and days on sick leave (6).

For example, in the USA, the cost of alcohol abuse along with all the consequences were around $249.0 billion in 2010 (7).

As for the total cost in the USA, it has been calculated that three-quarters of the costs of alcohol abuse are associated with binge drinking.

In the context of premature death and disability, concluded Lim and colleagues, alcohol misuse is listed as a fifth risk factor in general, but positioning to the first place among people between ages of 15 and 49 years (8). Also, approximately 25% of total number of deaths in the age group 20–39 years are associated with alcohol use, according to WHO (9).

Alcohol harmful use may have significant impact on social aspects and economic costs.

The WHO Global Status report on alcohol and health in 2014 reported:

The most prevalent tendency worldwide is an increase in recorded alcohol per capita consumption. This trend is mainly driven by an increase in alcohol consumption in China and India, which could potentially be linked to active marketing by the alcohol industry and increased income in these countries. The five-year trend in the WHO African Region, WHO European Region and, particularly, the WHO Region of the Americas is mainly stable, although some countries in the WHO European Region and the WHO African Region report significant decreases in alcohol consumption (1).

According to Anderson (1), there are five different intervention models considered to be efficacious based on the results of clinical trials: pharmacotherapy with counseling, cognitive behavioral therapy, motivational interviewing, and two models of brief interventions (1).

In different parts of the world, there are differences in the amount of drinks per capita. The dose of ingested alcohol also plays an important role with regard to the consequences. When it comes to injuries, the most important is the concentration of alcohol in the blood, while the chemical composition of alcoholic beverages affects the general state of health to a lesser extent (10).

Different societies and countries are taking legal measures in order to restrict the availability and use of alcohol, for example, limiting the size and/or increasing the price of beverages. Such measures are having an immediate, although limited effect (11).

As for the policy in this field, it is important to point out, according to research AMPHORA, that any limitation of alcohol consumption (permissible level of alcohol in the blood) leads to the reduction of the use of alcohol (12).

At the micro level: each reduction of the alcohol dose that is consumed, either by reducing the frequency of drinking or by reducing the quantity of alcohol consumed has an immediate impact on reducing the number of cardiovascular events and all kinds of injuries. This is particularly pronounced in heavy drinking.

As for the situation in Serbia, which is the central country of the Western Balkans, Djordjevic and his colleagues concluded that the pattern of drinking at a scale of 1–5 (the most risky form of drinking) is estimated at 3. As for the adolescent population in Serbia, 97.4% of them consumed alcohol and 34.9% of adolescents had the first experience with alcohol before 14 years of age, which coincides with the cultural pattern. Namely, in Serbia, children (especially male children) are allowed to try alcohol in a family environment, and it is considered a kind of test of manhood (13).

It is not surprising that the total social costs of alcohol abuse, both direct and indirect, are high. Direct costs are related to direct harmful effects of alcohol consumption (12). Indirect costs encompass loss of productivity, loss of quality of work, and so on. Even some studies show that the social costs (direct) are higher than medical costs, and indirect costs are even higher than the direct costs (14).

## Health Insurance

According to the World Health Organization alcohol consumption per capita is expected to increase up until 2025 (5). The largest increase is expected in the Western Pacific (dominantly in the Chinese population). Estimates are that growth will be about 1.5 l of pure alcohol per capita. The increase is expected in the Americas and South-Eastern Asia too (5). All these changes will certainly have significant repercussions not only on the financial burden but also on physical and mental health and narrower and broader social consequences.

From the perspective of payers, cost estimation is very important. The starting point for health economic analysis and decision making requires consideration of not only cost but also outcomes.

Most health insurance policies in the EU and USA will not cover all alcohol abuse treatments or rehab expenses. Typically, there are always some out-of-pocket costs.

It is a simple calculation, considering that health insurance covers a shorter hospital stay – the treatment usually remains on that level. Short hospitalizations are not sufficient to enable a patient to achieve a stable psycho-physical condition, so the level of health care that a patient receives does not correspond to their real needs. This just leads to poor treatment outcomes, frequent relapses, repeated hospitalizations, and finally, to more costs (11). Also, it usually means that new relapses in a short period of time. These and similar situations are cause for a new cycle of problems with insurance.

The questions concerning alcohol abuse or problems associated with the cost of treatment and insurance are evaluated periodically, both in professional circles and in the media. According to the sources listed in the report by http://HBO.com, many people are struggling with substance addiction, as well as with the lack of treatment because of insurance limitations (15).

In fact, everything that relates to extended treatment is not covered by insurance as well as rehabilitation programs.

According to information from the same source, the economic share of untreated alcohol dependence is $325 billion per year. Also, if a person is adequately treated (adequate therapy and length of treatment), $7–$12 on every dollar spent are saved (16).

Despite calculation and everyday clinical practice, patient’s real needs are ignored by medical and other professionals. At the end, patients are faced with a closed circle.

The National Health Insurance Fund organizes the health-care system for all citizens and permanent residents, but with obligations for employees, self-employed persons, and pensioners to contribute. The wealthier members of society need to participate with higher percentage of their income, based on a specified sliding scale. This method of financing is a residue of the socialist system of health insurance, but in recent years, this system has undergone serious changes (harmonization with EU law).

For example, research results show that the total allocation for health amounted to 8.13% of the gross domestic product (GDP) in 2003, whereas in 2014, it amounted to 9.91% of the GDP. The share of foreign donations in health financing decreased from 1.7% of the GDP in 2003 to 0.46% in 2014. Within the public sector funders of health care in Serbia, it has been found that the predominant funder was the Republican Health Insurance Fund (NHIF) with a share of 91.2% in 2003 and 93.99% in 2014 (15, 17).

Although the financial commitment by the NHIF increased, it eventually became insufficient because of increased costs incurred as a result of the growing needs of the population. The average patient (often with good reason) is dissatisfied with health care and the fact that they have to wait for an “ordinary” ultrasound a month or 2, and up to a year for a MRI, while for many drugs, they have to pay 90% of the original price.

In EU countries, national health systems face different kinds of major financial challenges (18).

## Situation with Life Insurance

Many insurance companies, for example, in the UK, treat alcoholism as a disease. Of course, there are certain limitations concerning insurance. According to WHO sources, the maximum level of alcohol intake per week is 21 U for men and 14 U for women (1). In any case, insurance companies paradoxically do not require medical examinations for people who consume more than 40 U a week (an alcoholic unit is equivalent to half pint of beer or one glass of wine). It is questionable whether this kind of model reduces total treatment costs of patients suffering from alcoholism or increases it (19).

The Medical Report or record from a GP contains all the important information about the health condition of the patient.

Section from the medical record considering occasional drinking provides information about person’s average consumption. Data about dependents and marital status are included into family section. Employer section provides information not only about current employment and person’s responsibilities at work but also regarding possible alcohol intrusion. The insurance companies in UK are not obliged to contact the employer. Persons who have been diagnosed with alcoholism and currently drinking have a less chance to achieve a life insurance cover. On the contrary, recovering alcoholic with a significant abstinent period can get a good life insurance quote. The abstinence of a year or 2 is usually enough for a person to get an average price of life insurance. Furthermore, new consequent relapses could also be considered as potential aggravating factor to accomplish this kind of financial benefits.

The General Assembly of United Nations (UN) adopted in 1991. A wide-ranging set of provisions entitled “Principles for the Protection of Persons with Mental Illness and for the Improvement of Mental Health Care.” According to Principle 4 of this important UN document (20, 21) (Table 1).

**Table 1:** **Principles for the protection of persons with mental illness and for the improvement of mental health care (UN, 1991)**.

A determination that a person has a mental illness shall be made in accordance with internationally accepted medical standards. A determination of mental illness shall never be made on the basis of political economic or social status or membership in a cultural, racial, or religious group or for any other reason not directly relevant to mental health status. Family or professional conflict, or non-conformity with moral, social, cultural, or political values or religious beliefs prevailing in a person’s community, shall never be a determining factor in the diagnosis of mental illness. A background of past treatment or hospitalization of a patient shall not of itself justify any present or future determination of mental illness. No person or authority shall classify a person as having, or otherwise indicate that a person has, a mental illness except for purposes directly relating to mental illness or the consequences of mental illness.

The stigmatization is more powerful than obeying the human rights. This applies to mentally ill individuals, or even more to addicts who are doubly stigmatized. In an attempt to initiate a more equitable access to treatment and affect inequality, in 2014, The International College of Person-Centered Medicine (the ICPCM) offered a new perspective of integrative health care oriented toward individuals and was formulated within the framework of the Geneva Declaration (20), which is as follows:

As global health challenges evolve, health care systems must include improved efforts to promote wellness, prevention, and effective chronic disease management. Health systems that fixate excessively on diseases and procedural interventions can lose sight of the person with the disease and the importance of that person’s life, family, and community. This has led to fragmentation of health care and has left most health care systems less effective and less efficient in achieving better population health outcomes. In many ways, the business of disease has overshadowed the provision of person-centered healthcare.

By analogy with the conclusion of a group of researchers proposed a new principle of health care, it can be said that all national health-care systems should be based on the principles of person-centered and community-based primary health care as a point of first contact of patients with the health-care system and the usual sources of patient care (20).

The heaviest burden of health risk and disease was observed in poor subpopulations of the poorest countries. The fact is that the people’s life chances varies not only because of poverty but also socioeconomic inequality. As part of the differentiation of society, numerous health problems that alcoholics have become more complicated as they go down the social scale (as they inevitably will, eventually), as well as more complex and more difficult to solve. Eventually, alcohol abuse leads to an escalation of anti-social behavior such as violence, drug abuse, criminal behavior, neglect and abuse of children, and to other problems including obesity, depression, and suicidal behavior. It is well known that if social inequality is present, bad outcomes (with regard to disease progression and consequences) can be expected both in individuals at the top and the bottom of the social hierarchy (22, 23).

## What Does it Mean?

Drinking alcohol in excess in any case represents a double risk (24).

Good intentions? Is it possible in the modern society where everything is subordinated to the welfare of the small number of people completely reversing the concept of disease and health, and to give everyone a chance to survive?

How can we do that? So far, the research (and interventions) focused on the effects on the population level, such as the increase in taxes, advertising bans, and the implementation of laws that prevent the use of alcohol in traffic. However, it seems that the problem may be viewed at the individual level.

In any case, the models of the treatment should be designed according to the needs of the individual. These models should incorporate not only the reduction of alcohol intake but also the path to abstinence. The plan should take into account the different (individual) needs for treatment, with regard to the degree of alcohol dependence and health status, and also include the needs of the family, community, and broader society.

## Author Contributions

MJ has developed main stream of ideas, has done literature search, and wrote the key theses. MA has done research and development of some of the basic theses.

## Conflict of Interest Statement

The authors declare that the research was conducted in the absence of any commercial or financial relationships that could be construed as a potential conflict of interest.
